# Noise-induced ribbon synapse loss in the mouse basal cochlear region does not reduce inner hair cell exocytosis

**DOI:** 10.3389/fncel.2024.1523978

**Published:** 2025-01-07

**Authors:** David Oestreicher, Alfonso Mauro Malpede, Annalena Reitmeier, Carolin Paula Bräuer, Laura Schoch, Nicola Strenzke, Tina Pangrsic

**Affiliations:** ^1^Experimental Otology Group, InnerEarLab, Department of Otolaryngology, University Medical Center Göttingen, Göttingen, Germany; ^2^Auditory Neuroscience Group, Max Planck Institute for Multidisciplinary Sciences, Göttingen, Germany; ^3^Auditory Systems Physiology Group, Institute for Auditory Neuroscience, InnerEarLab, University Medical Center Göttingen, Göttingen, Germany; ^4^Collaborative Research Center 889, University of Göttingen, Göttingen, Germany

**Keywords:** inner hair cell, noise trauma, ribbon synapse, isoflurane, calcium channel

## Abstract

Noise-induced hearing loss is one of the most common forms of hearing loss in adults and also one of the most common occupational diseases. Extensive previous work has shown that the highly sensitive synapses of the inner hair cells (IHCs) may be the first target for irreparable damage and permanent loss in the noise-exposed cochlea, more precisely in the cochlear base. However, how such synaptic loss affects the synaptic physiology of the IHCs in this particularly vulnerable part of the cochlea has not yet been investigated. To address this question, we exposed 3–4-week-old C57BL/6J mice to 8–16 kHz noise for 2 h under isoflurane anesthesia. We then employed hearing measurements, immunohistochemistry and patch-clamp to assess IHC synaptic function. Two noise sound pressure levels (SPLs) were used to evoke acute hearing threshold elevations with different levels of recovery 2 weeks post-exposure. Regardless of noise intensity, the exposure resulted in a loss of approximately 25–36% of ribbon synapses in the basal portions of the cochlea that persisted 2 weeks after exposure. Perforated patch-clamp recordings were made in the IHCs of the basal regions of the cochlea where the greatest synaptic losses were observed. Depolarization-evoked calcium currents in IHCs 2 weeks after exposure were slightly but not significantly smaller as compared to controls from age-matched non-exposed animals. Exocytic changes monitored as changes in membrane capacitance did not follow that trend and remained similar to controls despite significant loss of ribbons, likely reflecting increased exocytosis at the remaining synapses. Additionally, we report for the first time that acute application of isoflurane reduces IHC calcium currents, which may have implications for noise-induced IHC synaptic loss.

## Introduction

Disabling hearing loss is one of the most relevant common diseases, affecting more than 5% of the world’s population, with the number expected to rise continuously (World Health Organization, 2021). Sensorineural hearing impairment (HI) is caused by pathological changes in the auditory system or the vestibulocochlear nerve and can be either genetic or acquired. The latter may result from age-related degeneration, ototoxic drug exposure and noise-induced damage (Cunningham and Tucci, 2017). Noise-induced hearing loss (NIHL) is primarily linked to chronic exposure to high noise levels, commonly associated with noisy occupational environments, gunshots and blasts, the use of personal audio devices, and exposure during social settings such as nightclubs and concerts (Fink, 2024).

Excessive noise stimulation damages delicate structures in the inner ear, which can lead to temporary (e.g., minor stereociliar damage) or permanent changes (e.g., hair cell loss). Hair cell ribbon synapses have been recognized as very vulnerable elements of the cochlea (Kujawa and Liberman, 2009; reviewed in Liberman and Kujawa, 2017). Numerous animal models have shown that upon noise trauma, the number of ribbon synapses can remain permanently reduced without loss of hair cells and even after full recovery of elevated hearing thresholds (Furman et al., 2013; Hickman et al., 2020; Hickox et al., 2017; Kujawa and Liberman, 2009; Lin et al., 2011). Similar degeneration pathways with progressive neural deterioration in the aging cochlea that precedes hair cell loss were observed in animal-models studying age-dependent hearing loss (Fernandez et al., 2015; Parthasarathy and Kujawa, 2018; Sergeyenko et al., 2013) and in human temporal bone studies (Wu et al., 2019). It was furthermore shown that these degeneration processes are accelerated by noise exposure (Wu et al., 2021). The loss of synaptic ribbons leads to the loss of the associated spiral ganglion neurons (SGN) after months to years (Kujawa and Liberman, 2015). As a correlate, a permanent reduction in the amplitude of suprathreshold auditory brainstem response (ABR) wave I can be observed in the synaptopathic regions (Kujawa and Liberman, 2009). The neuronal loss is thought to primarily affect the low-spontaneous-rate fibers with high thresholds, as this would explain the recovered thresholds and the typical impairments associated with NIHL (Bourien et al., 2014; Furman et al., 2013; Liberman and Kujawa, 2017). Alternatively, a recent study in gerbils suggested that the initial loss of auditory fibers is not subtype-specific; rather, low-spontaneous-rate fibers may be less prone to recover as compared to high-spontaneous-rate fibers, effectively resulting in chronic loss of primarily low-spontaneous-rate fibers (Jeffers et al., 2021). Another study in CBA/CaJ mice did not find evidence for increased vulnerability of low-spontaneous rate fibers but revealed a new “hyperactive” subtype of auditory fibers with higher peak- and sustained rates after noise exposure (Suthakar and Liberman, 2021).

The mechanism underlying the loss of IHC ribbons is linked to neurotransmission at the IHC synapses, which are glutamatergic. Excessive glutamate release, as a result of acoustic overstimulation, can induce excitotoxic damage to the postsynaptic nerve terminals. In pharmacological experiments, perfusion with kainic acid leads to swelling and retraction of afferent cochlear nerve fibers (Pujol et al., 1985; Wan et al., 2014) and the genetically-induced absence of glutamatergic transmission or pharmacological blockade of Ca^2+^-permeable postsynaptic glutamate AMPA receptors prevents noise-induced loss of synaptic elements (Hu et al., 2020; Kim et al., 2019; Pujol et al., 1985; Sebe et al., 2017; Wan et al., 2014).

While two studies addressed early effects of noise exposure in the IHCs with temporary noise-induced synaptopathy in the apical turn of the cochlea with partially contradictory results on the effect of noise trauma on IHC synaptic function (Boero et al., 2021; Liu et al., 2019), it remains to be shown how the loss of ribbon synapses affects the notoriously more vulnerable basal IHCs of the organ of Corti. In our study, we established a mouse model of noise exposure under isoflurane gas anesthesia using two noise levels and recorded IHC synaptic activity in the basal turn of 5–6-week-old mice exposed 2 weeks prior to this measurement. We observed a moderate and delayed presynaptic ribbon and synapse loss that was not accompanied by reduced whole-cell exocytic activity, possibly suggesting upregulation of the synaptic activity at the remaining ribbons. Noise exposure under isoflurane anesthesia resulted in moderate temporary to mild permanent hearing loss. As previous studies described protective effects of isoflurane against noise trauma (Chung et al., 2007; Kim et al., 2005), but toxicity for ribbons in neonatal mice (Li et al., 2022), we tested its effects on the systems level, synapse density and also synaptic hair cell function in our experimental conditions. Prolonged isoflurane exposure resulted in a 5–8 dB shift of ABR thresholds, but left auditory synapse density unperturbed 2 weeks after exposure. This is in contrast to a prior study that detected a loss of SGNs after repeated isoflurane anesthesia in neonatal mice (Li et al., 2022). Using whole-cell patch clamp recordings of IHCs we observed an 18% reduction of voltage-gated calcium currents upon acute exposure to isoflurane. Reduced presynaptic calcium signaling and consequently glutamate transmission in the presence of isoflurane anesthesia might offer some protection of hair cells and ribbon synapses against noise-induced damage, but cannot completely prevent it.

## Materials and methods

### Animals

Male C57BL/6J mice at the age of 3–4 (D0) and 5–6 (D14) weeks were used for electro-physiological, immunohistochemical and systems physiology experiments. All experiments complied with the national animal care guidelines and were approved by the University of Göttingen Board for animal welfare and the animal welfare office of the state of Lower Saxony.

### Systems physiology recordings: auditory brainstem responses (ABRs) and distortion product otoacoustic emissions (DPOAEs)

Mice were anesthetized using isoflurane (5% Induction/2% Maintenance). Recordings were obtained in a custom-made soundproof box (IAC GmbH, Germany) with a Tucker-Davis (TDT) BioSig System III/BioSig software (Tucker-Davis Technologies, Alachua, FL). Body temperature was maintained using a heating pad (37°C) and ECG was monitored continuously. For ABR recordings, subcutaneous needles were placed at the vertex, the ipsilateral pinna and at the lower back. Using a JBL2402 speaker in free-field configuration, tone bursts (10-ms plateau, 1-ms cos2 rise/fall) were ipsilaterally presented at 19 Hz. ABR traces were amplified 10.000 times and filtered from 400 to 4.000 Hz, to obtain two separate mean ABR traces and averaged from 1,000 repetitions. Thresholds were defined in stacked waveforms as the lowest stimulus intensity (dB SPL) at which a reproducible waveform could be visually detected with a 5 dB precision. Data from animals where fewer than four frequencies were measured reliably were excluded from the analysis. DPOAEs were measured using a custom-written MATLAB (MathWorks) routine. Two primary tones f1 and f2 (ratio f1/f2 = 1.2, level difference f2 = f1 + 10 dB) were presented via two speakers (MF-1, TDT) and a custom-made ear probe containing an MKE-2 microphone (Sennheiser). Stimulus duration was 16 ms and f2 levels varied from 10 to 70 dB SPL in 5 dB steps. For higher intensities, we extrapolated a threshold. The microphone signal was amplified (UAC-2; Zoom) and digitized (TDT System). DPOAE amplitude was analyzed at 2 × f2-f1 using custom-written MATLAB software with fast Fourier transformation. DPOAE thresholds were determined as the interpolated f1 intensity at which the DPOAE intensity exceeded −5 dB SPL.

### Noise exposure

The animals were exposed to an 8–16 kHz band noise for 2 h at 92 or 96 dB (SPL). Noise exposure was performed in a custom-made soundproof box (IAC GmbH, Germany) under isoflurane gas anesthesia (5% Induction/2% Maintenance) for the sake of animal welfare and refinement of animal experiments. The noise waveform was created digitally using a TDT System III controlled by a custom-written Matlab routine that was driving a JBL2402 speaker to generate an 8–16 kHz noise band with a flat frequency spectrum.

### Patch-clamp

For patch-clamp recordings, the basal turn of the cochleae was dissected in Modified Ringer’s solution (MRS) containing (in mM): 111 NaCl, 35 TEA-Cl, 2.8 KCl, 1 MgCl_2_, 1 CsCl, 10 NaOH-HEPES, 1.3 CaCl_2_, and 11.3 D-glucose (pH 7.2, 305 mOsm/L). IHCs of the ∼48 kHz tonotopical region were investigated using the perforated patch configuration. The pipette solution contained (in mM): 130 Cs-gluconate, 10 TEA-Cl, 10 4-AP, 1 MgCl_2_, 10 HEPES, 300 mg/mL amphotericin B, pH 7.2 ∼ 290 mOsm. MRS was also used for the bath perfusion. Recordings were performed at room temperature using an EPC-10 amplifier (Heka-Germany) controlled by Patchmaster software. Currents were leak-corrected using a p/10 protocol, sampled at 50 kHz and corrected offline for the liquid junction potential (−14 mV). Only recordings showing leak currents lower than 50 pA were included in the final analysis.

For *in vitro* isoflurane experiments a 0.6 mM isoflurane solution [close to 2 MAC (the minimum alveolar concentration required to suppress movement in response to noxious stimulation in 50% of subjects) for isoflurane in mice (Sonner et al., 2007); 0.31 mM corresponds to 1 MAC for isoflurane in mice] was prepared from a saturated stock solution in gas-tight containers as described earlier (Baumgart et al., 2015). Superfusion of 0.6 mM isoflurane was carried out with a perfusion system connected to a manifold tip with a diameter of 200 μm (ALA QTP-200, ALA Scientific Instruments, United States). Recordings were initiated 3–5 min after perfusion initiation. These were compared to the recordings prior to isoflurane application and during wash-out.

### Immunohistochemistry and confocal microscopy

Cochleae were fixed in 4% formaldehyde (FA) for 10 min on ice and decalcified overnight in EDTA (0.5 M, pH 8) at 4°C. After permeabilization in 1% Triton X-100, cochleae were blocked in goat serum dilution buffer (GSDB; 16% normal goat serum, 450 mM NaCl, 0.3% Triton X-100, and 20 mM phosphate buffer at pH 7.4) for 1 h on ice and incubated in primary antibodies in GSDB with chicken anti-Homer1 (Synaptic Systems, 160,006), mouse anti-CtBP2 (BD Transduction Laboratories, 612,044) and rabbit anti-Myo6 (Proteus Biosciences, 25–6,791) at 4°C overnight. This was followed by a second fixation of 2 h in 4% FA. After that, organs of Corti were dissected into 3–4 pieces and incubated with matching Alexa-fluorophore conjugated secondary antibodies for 1 h at room temperature. Explants were then mounted in Mowiol mounting medium.

Synapse counts: Images to determine regions of interest were acquired with a Zeiss LSM 510 META confocal microscope and a 10× air objective. A tonotopic map for each individual organ of Corti was calculated using a custom-made Matlab (Mathworks) routine based on the mouseline-specific total length of the organ of Corti (Müller et al., 2005). For the areas of interest (8, 16, 24, 32, and 48 kHz) z-stacks were acquired with an Abberior Expert Line confocal microscope using a 1.4 NA 100× oil-immersion objective, 80 × 80 nm pixel size and a z-step size of 200 nm.

Hair cell counts: For hair cell counts images were acquired with a Leica SP8 confocal microscope using a 1.4 NA 20× oil-immersion objective, 378.8 × 378.8 nm pixel size and a z-step size of 0.65 μm. Images were taken from the apical (8–12 kHz) and the basal turn (32 kHz region) of each analyzed organ. OHCs were counted and divided by the length of the analyzed region (222 ± 10 μm).

### Ribbon counts

Confocal immunofluorescence images were adjusted for brightness and contrast using ImageJ (NIH) and analyzed using Imaris (Bitplane) software. Pre- and postsynaptic immunofluorescent spots were detected by the spot detection tool of Imaris in a region of interest of about 6–10 IHCs. The number of synapses was analyzed by counting juxtaposed immunofluorescent spots labeling presynaptic ribbon constituent CtBP2 and a postsynaptic density constituent Homer1.

### Data analysis

Electrophysiological data were analyzed using custom analysis routines written in Igor Pro Software (Wavemetrics). ABR and DPOAE recordings were analyzed by custom-written Matlab (Mathworks) scripts. All data are presented as mean ± SEM. Data were tested for significance using ANOVA with Dunnett’s multicomparisons test to detect possible statistically significant differences to the respective control. In case of *in vitro* isoflurane effects, Tukey’s multicomparisons test was chosen to allow comparisons between data acquired before and upon isoflurane application as well as during the wash-out phase.

## Results

### Noise exposure under isoflurane anesthesia results in moderate acute increase of ABR and DPOAE thresholds with almost complete or partial recovery at two noise levels

**Figure 1:**
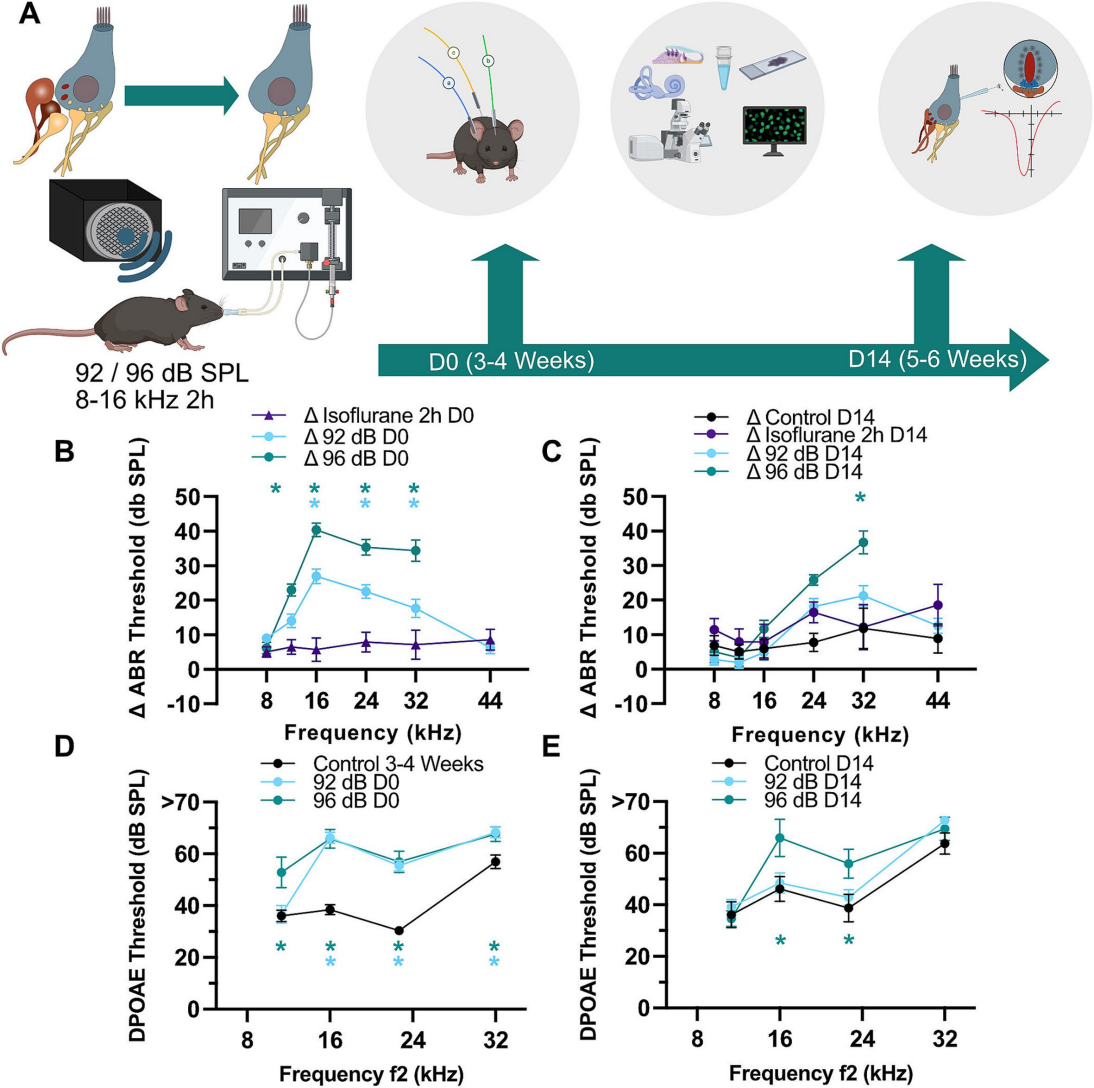
Partial recovery of threshold shifts and otoacoustic emissions after moderate noise trauma. **(A)** To establish a noise-induced auditory synaptopathy in the basal turn of the cochlea male C57/Bl6 mice of 3–4 weeks of age (D0) were exposed to a white noise from 8 to 16 kHz with 92 or 96 dB SPL for 2 h under isoflurane gas anesthesia. Hearing was tested (ABR, DPOAE) before and after exposure and in a subset of animals 2 weeks after exposure. The final hearing measurement was followed by histological and/or cell physiological assessment of IHC synapses. Partially created with BioRender. **(B)** ABR threshold shifts immediately after exposure as compared to pre-exposure values obtained in the same animals. Immediately after noise exposure to either sound pressure level (D0; 92- and 96-dB) the ABR shifts were significantly higher as compared to the shift caused by isoflurane anesthesia alone (asterisks: Dunnett’s multiple comparisons test; *p* < 0.001 for 96-dB at 12 kHz, <0.002 and <0.03 for 92-dB at 24 and 32 kHz, respectively; <0.0001 for other asterisks). *N* = 31, 32, 31, 32, 32, 25 for the 92-dB D0 group at 8–44 kHz frequencies, respectively. *N* = 7 for the D0 Isoflurane 2 h group at all tested frequencies. *N* = 17, 17, 15, 15, 15 for the 96-dB D0 group tested at 8–32 kHz. **(C)** ABR thresholds showed better recovery in the 92- as compared to 96-dB group (asterisk: Dunnett’s multiple comparisons test; *p* = 0.0001). *N* = 25, 26, 25, 26, 25, 18 for the 92-dB D14 group. *N* = 7 for the D14 Isoflurane 2 h group at all tested frequencies. *N* = 11, 11, 11, 11, 11, 8 for the non-exposed control. *N* = 6 for the 96-dB D14 group tested at frequencies 8–32 kHz. **(D,E)** DPOAE thresholds remained significantly elevated at 16 and 22.7 kHz for 96 dB after 14 days (D14) (Dunnett’s multiple comparisons test; *p* < 0.02). *N* = 16 for Control D0; *N* = 14 for 92-dB D0 group; *N* = 13 for 96-dB D0 group. *N* = 9 for Control D14; *N* = 13 for 92-dB D14 group; *N* = 6 for 96-dB D14 group at all tested frequencies. *p*-values for asterisks in **(D)**: <0.0001 at 16 and 24 kHz for both noise levels, 0.0002 (96-dB group at 8 kHz), 0.01 and 0.02 at 32 kHz for 92- and 96-dB group, respectively.

To investigate the effects of a two-hour exposure to 8–16 kHz noise band under isoflurane anesthesia at 92 and 96 dB SPL, we assessed auditory function immediately after (D0) and 14 days post-exposure (D14) by measuring auditory brainstem responses (ABR) and distortion product otoacoustic emissions (DPOAE) (Figure 1). We observed an acute ABR threshold shift of around 20 dB following an exposure to 92 dB SPL and 40 dB upon exposure to 96 dB SPL noise (Supplementary Figures S1A,B). Since gas anesthesia through isoflurane was shown to elevate hearing thresholds (Kim et al., 2012; Sheppard et al., 2018) and protect hair cells against noise-induced permanent auditory damage accompanied by hair cell loss (Kim et al., 2005) we evaluated a possible influence of isoflurane on the recorded hearing thresholds and noise exposure. For this, we performed an additional set of experiments, in which animals were kept under identical isoflurane anesthesia conditions but in the absence of noise and similarly recorded hearing threshold before and immediately after 2-h isoflurane exposure (Isoflurane 2 h D0) as well as 2 weeks afterwards (Isoflurane 2 h D14). Under these experimental conditions, we observed an acute ABR threshold shift of on average 5–8.5 dB SPL across the measured frequencies (Figure 1B; Isoflurane 2 h D0), while DPOAE amplitudes remained unaffected by prolonged isoflurane exposure (Supplementary Figure S1C). We then calculated the threshold shifts of ABR measurements conducted before and after noise exposure and compared them to threshold shifts of the Isoflurane 2 h D0 group. This revealed significantly larger ABR threshold shifts upon exposure to 92- and 96-dB noise bands as compared to the shifts observed with isoflurane exposure alone [Figure 1B; Dunnett’s test; *p* < 0.05 (see also figure legends): Δ92 dB D0 vs. ΔIsoflurane 2 h D0 at 16–32 kHz; Δ96 dB D0 vs. ΔIsoflurane 2 h D0 at 12–32 kHz; ΔIsoflurane 2 h D0 *N* = 7, Δ92dB D0 *N* = 32, Δ96dB D0 *N* = 17]. Two weeks after exposure, ABR thresholds largely recovered in the 92-dB group, but remained elevated at 32 kHz in the 96-dB group (Figure 1C; Dunnett’s multiple comparisons test; *p* = 0.0001 for Δ96 dB D14 vs. ΔIsoflurane 2 h D14 at 32 kHz; ΔIsoflurane 2 h D14 *N* = 7, Δ92 dB D14 *N* = 26, Δ96 dB D14 *N* = 6).

A smaller but also significant effect was observed in DPOAE thresholds, which were similarly raised in both exposure groups and report outer hair cell (OHC) malfunction likely related to acute damage of stereocilia [Figure 1D; Dunnett’s multiple comparisons test; *p* < 0.05: 92 dB D0 vs. Control D0 at 16–32 kHz; 96 dB D0 vs. Control D0 at 11.3–32 kHz (see also figure legends); Control D0 *N* = 16, 92 dB D0 *N* = 14, 96 dB D0 *N* = 13]. Two weeks later, DPOAEs of exposed animals were undistinguishable from age-matched control animals in both groups except at 16 and 22.7 kHz for 96-dB (Figure 1E; Dunnett’s multiple comparisons test; *p* < 0.02: 96 dB D14 vs. Control D14 for 16 kHz and 22.7 kHz; Control D14 *N* = 9, 92 dB D14 *N* = 13; 96 dB D14 *N* = 6).

**Figure 2:**
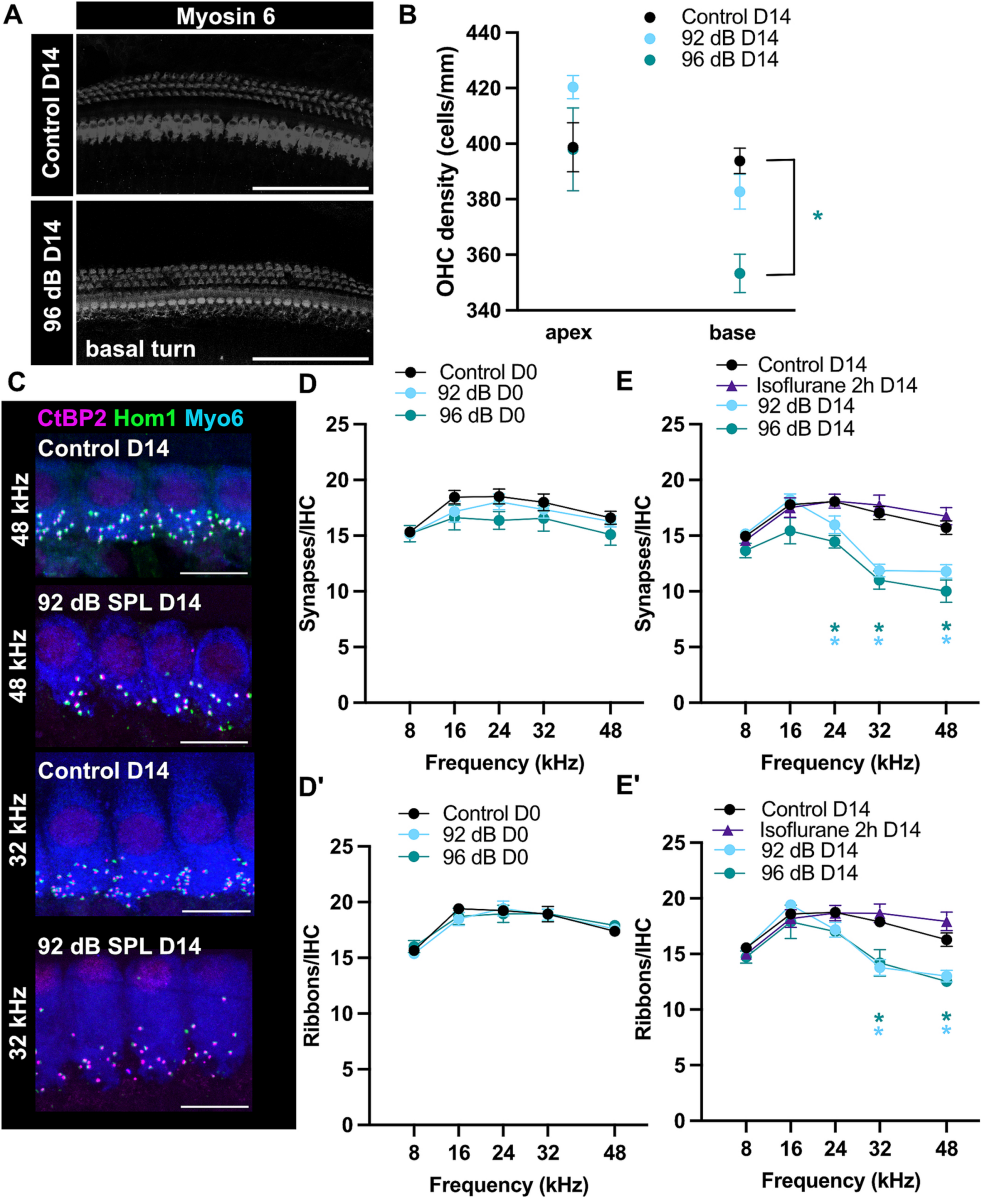
Ribbon-synapse loss is persistent but delayed. **(A)** Examples of maximum-projections of confocal z-stacks showing myosin 6-immunostained hair cells 2 weeks after 96-dB noise-trauma and in age-matched controls. Scale bars: 100 μm. **(B)** Reduced OHC-density was detected in the cochlear base of the 96-dB (Dunnett’s test; *p* < 0.05), but not 92-dB group (apex: *N* = 6, 3, 6; base: *N* = 6, 7, 5 for Control D14, 92 dB D14 and 96 dB D14, respectively). **(C)** Maximum-projections of confocal z-stacks depicting IHCs from a noise-traumatized and control animal in the 48- and 32-kHz-region. Scale bars: 10 μm. Acutely after noise-exposure there was no significant change in either synapse **(D)** or ribbon **(D′)** density (Control D0 *N* = 13, 13, 12, 11, 12 for the respective frequencies; 92 dB D0 *N* = 8, 8, 8, 7, 6; 96 dB D0 *N* = 7, 7, 7, 6, 6 for the frequencies 8–48 kHz). However, note a trend to reduced numbers of IHC synapses. **(E)** Fourteen days after noise exposure ribbon density was significantly reduced at 32–48 kHz and **(E′)** synapse density at 24–48 kHz (Dunnett’s test; *p* < 0.05; see Supplementary Table S1; Control D14 *N* = 17, 18, 17, 18, 12; 92 dB D14 *N* = 11, 11, 14, 18, 15; 96 dB D14 *N* = 5, 5, 3, 6, 2; Isoflurane 2 h D14 *N* = 4, 6, 6, 6, 5). Prolonged isoflurane exposure in the absence of noise trauma caused no synaptic loss.

Increased DPOAE (and concomitantly ABR) thresholds 2 weeks post-exposure could be related to persistent stereocilia damage or hair cell loss (Kurabi et al., 2017). To test for potential cell loss, we performed immunohistochemistry of the organs of Corti and counted the number of OHCs in the basal part of the cochlea (32 kHz area) of exposed and age-matched control animals 2 weeks after exposure (Figures 2A,B). We detected decreased OHC density in the animals exposed to 96-dB, but not 92-dB noise band (Figure 2B; Dunnett’s multiple comparisons test, *p* < 0.05). A partial loss of the OHCs in the 96-dB group can explain the reduction of high frequency hearing sensitivity (increased DPOAE and ABR thresholds) 2 weeks post-exposure. Increased threshold at 16 kHz in the 96-dB group at 2 weeks after exposure, on the other hand, is hard to reconcile with the observation of no significant increase in the ABR threshold in this frequency region. We postulate that due to the nature of the underlying stimulus the effect in the DPOAE may be detected more precisely. With this respect, it is worth noting that a discrepancy between the DPOAE and ABR thresholds was observed in the past in clinical studies, where DPOAEs proved superior to pure tone audiometry as a screening tool to detect hair cell malfunction: DPOAE amplitudes first decreased before threshold changes were detected at the corresponding frequencies in pure tone audiometry in patients that received cisplatin treatment or in a cohort of hemodialyzed patients. Reduced emissions with normal hearing could therefore indicate an underlying pathological condition that may later lead to significant hearing loss (Bendo et al., 2015; Knight et al., 2007; Ress et al., 1999).

### Permanent loss of hair cell ribbon synapses after moderate noise trauma under isoflurane anesthesia

We assessed synapse density of specific tonotopic regions by counting ribbons and postsynaptic boutons through colocalizing anti-CtBP2 and anti-Homer 1 immunospots per IHC, respectively (Figures 2C–E′). In control, non-exposed ears, the mean counts showed a broad peak of around 19 ribbons/IHC in mid-cochlear regions, slightly declining toward the apical and basal ends (Figures 2D–E′). These values are consistent with previously reported data (Kim et al., 2019; Kujawa and Liberman, 2009; Meyer et al., 2009).

D0 organs of Corti were fixed immediately after a hearing test following the two-hour noise exposure and immunostained in the following days. Within 2 h of noise trauma and shortly thereafter there were no evident signs of synaptic loss in either the pre-synaptic or post-synaptic elements (Dunnett’s multiple comparisons test; see Supplementary Table S1), suggesting that in our recording conditions the synaptic degeneration (e.g., disassembly of the ribbons or loss of boutons or postsynaptic density proteins) does not occur during noise exposure or immediately thereafter but develops over the next days following exposure. In a subset of experiments, cochleae of the 92-dB-noise group were harvested 1 day post-exposure (D1). These revealed a significant loss of 17–19% of ribbons and 25–30% of ribbon synapses (t.i. juxtaposed CtBP2/Homer1 immunospots) in the high-frequency regions (32–48 kHz) (Supplementary Figure S2; Dunnett’s test; Control D0 vs. 92 dB D1: *p* < 0.01 for 32 and 44 kHz; 92 dB D1 *N* = 5). Synaptopathy at D1 however was not yet fully developed. Two weeks after exposure, a synaptic loss of an approximately 20–23% of ribbons and 25–36% of ribbon synapses was observed in the high-frequency regions (≥32 kHz) of the noise-exposed ears for both noise intensities examined (92- and 96-dB SPL) and to a lesser extent at 24 kHz 2 weeks after exposure (Figures 2E,E′). Furthermore, in the high-frequency regions on average one to two more orphan ribbons were observed per IHC of exposed animals 2 weeks after exposure, which is comparably higher to studies exposing CBA or C57Bl/6J mice in awake state (Fernandez et al., 2015; Kim et al., 2019; Liberman et al., 2015). Interestingly, the stronger noise exposure did not cause significantly more synaptic loss as compared to the lower sound intensity, but lead to larger “permanent” shift in the ABR and DPOAE thresholds (Figure 1). In the high-frequency (32–48 kHz) regions of the noise-exposed ears, the ribbon density was observed to decrease to approximately 13/IHC 2 weeks after exposure. Conversely, in the lower-frequency (≤24 kHz) regions, no alterations in the ribbon density were observed (Figure 2E′). Previous studies suggested that prolonged exposure to volatile gas anesthetics alone can result in loss of a fraction of ribbon synapses in the cochleae of newborn mice (Li et al., 2022). To test whether the observed loss of synapses in our exposed animals may have been caused by isoflurane exposure, ribbon synapse density was assessed in the organs of Corti of animals under prolonged isoflurane anesthesia (Figures 2E,E′). We observed no difference to the control group (organs of Corti from non-exposed animals that did not undergo prolonged isoflurane anesthesia). This suggests that the observed synaptic loss in the noise-exposed groups is indeed caused by noise exposure and a single prolonged exposure of 3-week-old mice to low levels of isoflurane (2% vol.) does not cause obvious cochlear synaptic loss within 2 weeks after exposure to the anesthetic.

### Noise-induced ribbon loss does not result in decreased IHC exocytosis

**Figure 3:**
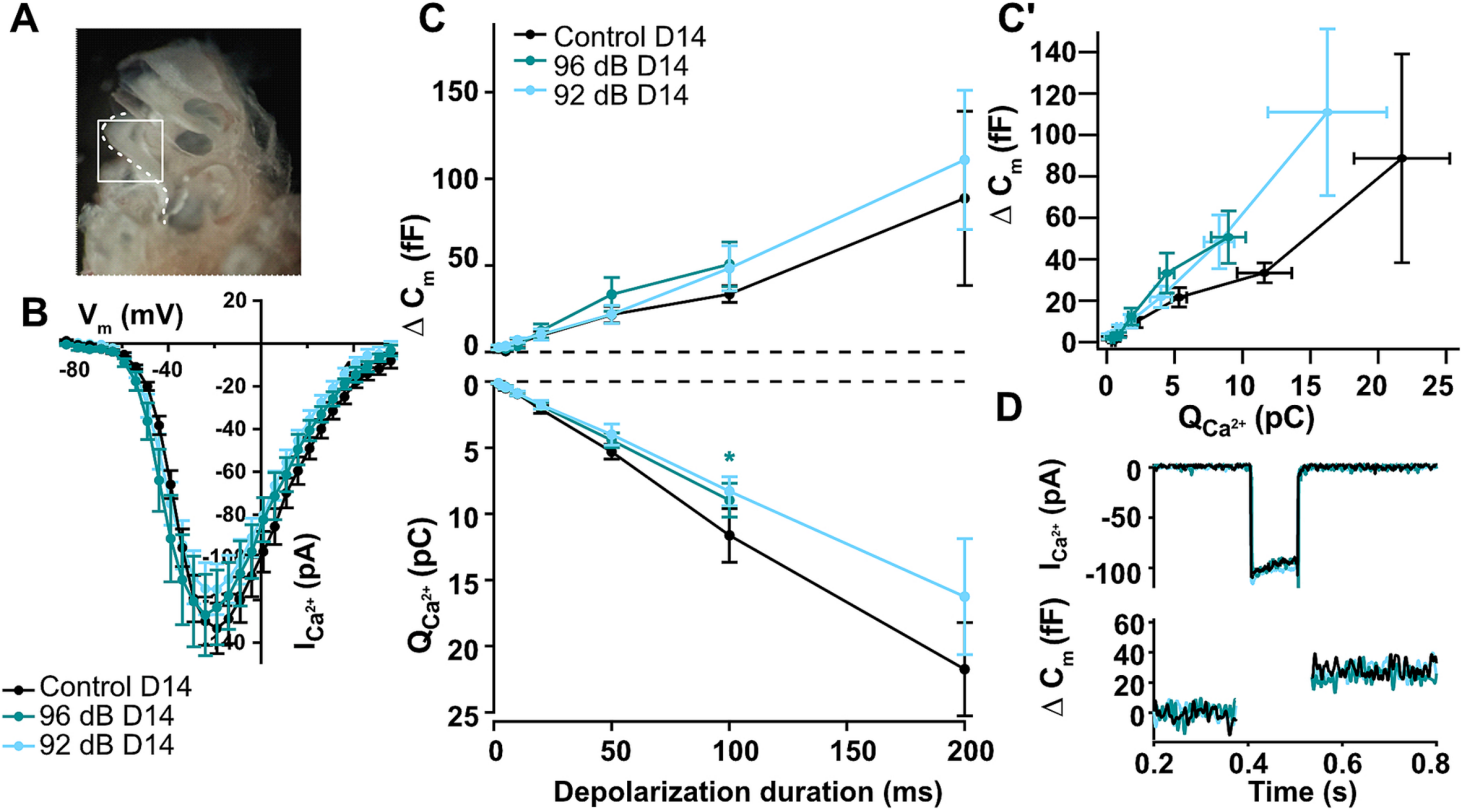
Noise-induced auditory synaptopathy in the basal turn of the cochlea leaves overall IHC synaptic activity largely unchanged. **(A)** An image of the mouse cochlea with exposed cochlear turns and region that was extracted (white) with the basal turn that was used for the patch-clamp measurements of high-frequency IHCs after noise-trauma. **(B)** Current–voltage-relationships recorded 2 weeks after noise exposure show comparable Ca^2+^ current amplitudes between noise-exposed and unexposed controls (Control D14 *n* = 9, 96 dB D14 *n* = 5, 92 dB D14 *n* = 9). **(C,C′)** Calcium-dependent exocytosis as measured by increments of membrane capacitance was not significantly different from control hair cells 14 days after noise trauma for any of the noise intensities, but a slight trend toward increased efficiency of exocytosis was detected (Control D14 *n* = 3, 4, 6, 7, 9, 13, 5; 96 dB D14 *n* = 3, 4, 5, 5, 8; 92 dB D14 *n* = 6, 9, 9, 10, 10, 13, 6). Asterisk: *p* < 0.03; none of the other points showed significant differences (Dunnett’s tests for dCm and QCa; *p* > 0.05). The efficiency of the sustained exocytosis (dCm/QCa at 20–100 ms) showed a trend toward increased values for the noise-exposed IHCs (two-way ANOVA: *p* = 0.0505; but none of the individual points reached statistical significance). **(D)** Representative calcium-current and capacitance traces upon a 100-ms long depolarization to maximal calcium current potential.

Patch-clamp measurements from noise-exposed apical IHCs revealed mostly comparable levels of presynaptic activity upon short to mid-long depolarization steps to age-matched non-exposed controls despite temporary loss of synaptic ribbons (Boero et al., 2021). However, a prior study published conflicting results demonstrating significantly reduced exocytosis after temporary ribbon loss in 4-week-old mice (Liu et al., 2019). To directly test the effects of noise exposure in the IHCs of the most vulnerable, basal part of the cochlea (Figure 3A), we performed these challenging recordings from a subset of our exposed organs of Corti. Whenever possible, the same animals were used to assess the synaptic density as well as IHC synaptic physiology to obtain most comparable results. Current–voltage relationships in the basal IHCs of 5–6-week-old mice revealed similar maximal currents (Figure 3B) but slightly larger membrane capacitance responses as previously observed in the adult basal IHCs of the gerbils or mice (Johnson and Marcotti, 2008; Zuccotti et al., 2012). Surprisingly, patch-clamp recordings 2 weeks after noise exposure showed no significant changes in IHC synaptic function on the whole-cell level (Figure 3C, upper panel; Dunnett’s multicomparison test; example traces in Figure 3D) despite a moderate but significant decrease in ribbon density at high-frequency tonotopic positions (20–23%), as identified by immunofluorescence (Figure 2). The relative amount of membrane capacitance change upon a 100-ms depolarization step thus increased from approx. 2.1 fF/ribbon in control to 3.7 fF/ribbon (by 81%) and to 4.0 fF/ribbon (by 97%) in the IHCs exposed to 92- and 96-dB, respectively. Calcium charge transfer was slightly but not significantly reduced upon noise exposure (Figure 3C, lower panel; apart from the 100-ms depolarization pulse in the 96-dB group; *p* < 0.03), which is consistent with the observation of no significant reduction in the maximal calcium current amplitudes at short depolarization pulses (Figure 3B). Observation of a modest decrease in the calcium charge transfer together with the slight (but non-significant) increase in the whole-cell membrane capacitance jumps prompted us to test the efficiency of sustained component (≥20-ms pulse duration) of exocytosis (Figure 3C′). A trend toward an increased efficiency of exocytosis upon noise exposure was observed (two-way ANOVA; *p* = 0.0505), which however did not reach statistical significance at any of the individual observation points (Dunnett’s multicomparison test, *p* > 0.05). Together, these observations suggest that the remaining (ca. 80%) ribbon synapses may undergo compensatory mechanisms to maintain overall synaptic functionality. This is consistent with the study of Boero et al. (2021), in which maintained exocytosis for shorter- and a significant increase in IHC exocytosis for very long depolarization durations was observed 1 day after exposure of pre-weaning animals. This potentiation of exocytosis was suggested to be driven by glutamate-dependent mechanisms (Boero et al., 2021).

### Acute effects of isoflurane on IHC physiology

**Figure 4:**
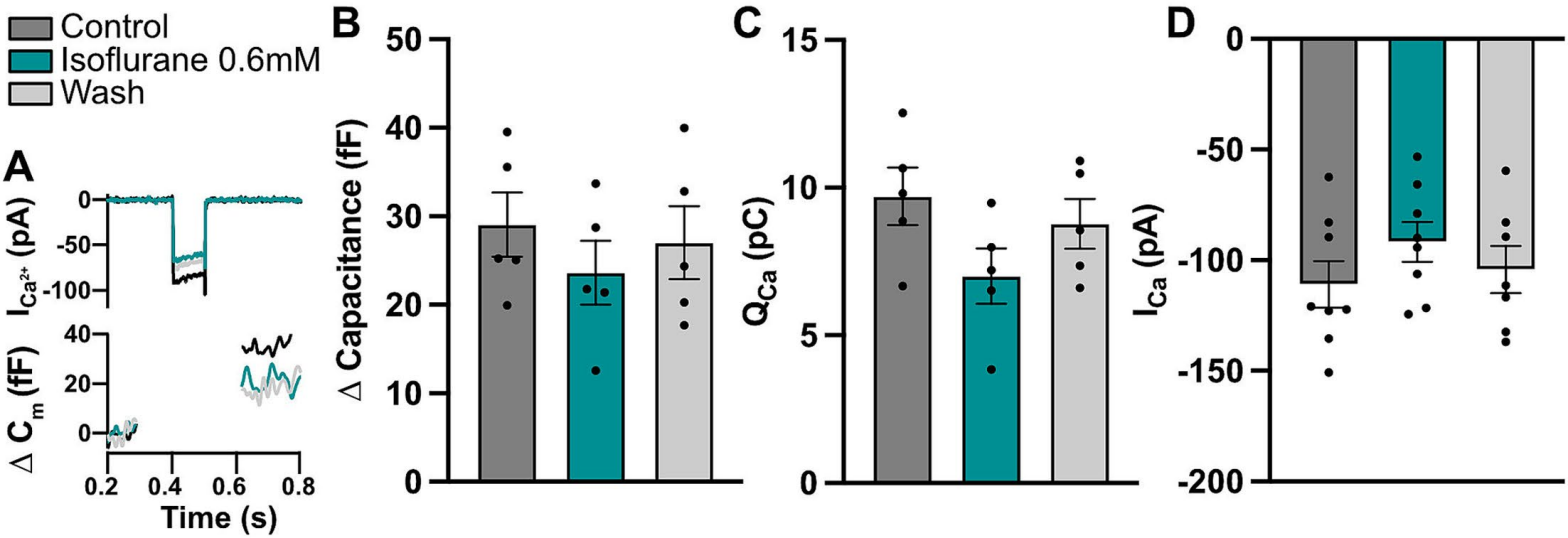
Isoflurane affects IHC calcium currents. **(A)** Representative calcium current and capacitance traces upon a 100-ms step depolarization step to maximal current potential for the different conditions. **(B)** A trend toward decreased exocytosis upon a 100-ms step depolarization could be observed in acutely-exposed IHCs (one-way ANOVA with Tukey’s multiple comparison, *p* > 0.05; *n* = 5). **(C)** Calcium charge transfer was significantly reduced during isoflurane perfusion, but recovered after wash-out (one-way ANOVA with Tukey’s multiple comparison, for *p* values see text; *n* = 5). **(D)** Calcium current amplitudes upon 10-ms long step depolarizations to the maximum calcium current potential were significantly reduced upon isoflurane application and recovered during wash-out (one-way ANOVA with Tukey’s multiple comparison, for *p*-values see text; *n* = 8).

As shown in this and previous studies, acute exposure to isoflurane increases ABR thresholds (Figure 1) and reduces ABR wave I amplitudes, likely due to recruitment of fewer auditory nerve fibers (Cederholm et al., 2012). The exact action of isoflurane on cochlear function is not entirely understood, but may involve direct effects on hair cell function. For example, isoflurane was shown to inhibit neurotransmitter release in hippocampal neurons (Herring et al., 2009; Li et al., 2022) and modulate the function of voltage-gated Na^+^ and Ca^2+^ channels (Baumgart et al., 2015; Herold et al., 2017; Herring et al., 2009; Westphalen et al., 2011). To understand the extent of putative acute isoflurane exposure effects on IHC synaptic physiology, we employed bath perfusion of 0.6 mM isoflurane [corresponding to 1.935 MAC for isoflurane in mice (Sonner et al., 2007; see methods)] and performed patch-clamp experiments in IHCs of acutely isolated organs of Corti. Here, IHCs from the apical turns of the organs were used due to much better accessibility to electrophysiological recordings. Calcium currents upon short depolarizations to different potentials as well as membrane capacitance changes upon 100-ms long depolarization steps to the maximal calcium current potential were recorded during perfusion of normal saline containing 1.3 mM [Ca^2+^], upon application of isoflurane and consequent wash-out (Figure 4). In our recording conditions, the perfusion of 0.6 mM isoflurane typically took maximal effects within 3–5 min and caused an approximately 18% reduction in the maximal calcium currents (Figure 4D; one-way ANOVA with Tukey’s multiple comparison: Control vs. Isoflurane and Isoflurane vs. Wash out *p* < 0.01; Control vs. Wash out *p* > 0.05; *n* = 8). Similarly, we observed a significant reduction in the calcium charge transfer upon 100-ms long depolarization steps (Figures 4A,C; one-way ANOVA with Tukey’s multiple comparison: Control vs. Isoflurane *p* < 0.0001; Control vs. Wash out *p* > 0.05; Isoflurane vs. Wash out *p* < 0.05; *n* = 5). This resulted in modestly reduced membrane capacitance jumps, which however did not reach statistical significance, likely due to larger variability (Figure 4B; one-way ANOVA with Tukey’s multiple comparison, *p* > 0.05 for all comparisons, Control vs. Isoflurane; Control vs. Wash out; Isoflurane vs. Wash out; *n* = 5). *In vivo*, reduced IHC presynaptic calcium signaling and consequently glutamate transmission in the presence of isoflurane anesthesia might offer some protection of hair cells and ribbon synapses against noise-induced damage, but as demonstrated in the current study, cannot completely prevent it.

## Discussion

In this study we show delayed moderate auditory synaptopathy with acute moderate ABR threshold shift that largely recovers upon noise exposure (2-h, 8–16 kHz noise band) under isoflurane anesthesia in young weaned C57Bl/6J male mice. Increasing the sound pressure level from 92 to 96 dB led to a permanent threshold shift at high frequencies, but no further increase in synaptic loss, which amounted to about (11-) 25–36% in the (mid to) basal tonotopic regions of the cochlea. The loss of paired pre- and post-synaptic structures scaled with the loss of presynaptic ribbons, with few orphan ribbons detected throughout analyzed organs. The loss of ribbons was accompanied by slightly, but not significantly reduced IHC whole-cell calcium currents and no reduction in IHC exocytosis, possibly suggesting long lasting compensatory mechanisms on the whole-cell level. Our data also suggests that isoflurane reduces IHC synaptic calcium currents. Hence, reduced hair cell synaptic activity in the presence of isoflurane anesthesia may partially protect ribbon synapses from noise exposure possibly explaining the relatively modest synapse loss and modest permanent ABR threshold shift upon 96-dB noise trauma.

### Noise-induced ABR and DPOAE thresholds measured under isoflurane anesthesia

Immediately after noise exposure, the mice showed 10–40 dB SPL threshold shift in the ABRs at frequencies ≥16 kHz. To account for the threshold shift caused by anesthesia alone, we compared the ABR threshold shifts upon 2-h noise exposure under anesthesia to the shifts produced by 2-h isoflurane anesthesia alone, in the absence of acoustic overstimulation. Based on these comparisons, we conclude that a 2-h noise exposure at 92 dB SPL caused a transient threshold shift with an almost complete recovery within the following 2 weeks. Acute elevation of ABR hearing thresholds was relatively modest, which we ascribe to protective effects of isoflurane, effectively reducing the amount of cochlear activation. Increasing noise exposure to 96 dB SPL resulted in more extensive temporary loss of hearing sensitivity and mild permanent threshold shift, as observed 2 weeks after exposure at high frequencies. Traditionally, OHCs were considered the most vulnerable cochlear elements that may become damaged and lost upon noise trauma resulting in a permanent hearing threshold shift (Kurabi et al., 2017). To assess the functionality of OHCs in our system, we measured DPOAE thresholds. Immediately post-exposure, we observed a significant DPOAE threshold shift, which completely recovered for the 92-dB but not 96-dB group 2 weeks after exposure. Thus, mild permanent threshold shifts as detected in the ABRs upon 96-dB noise exposure was likely connected to OHC damage, as also suggested by the observation of partial loss of basal OHCs.

Isoflurane elevates ABR thresholds as demonstrated across various species (Cederholm et al., 2012; Ruebhausen et al., 2012; Stronks et al., 2010). In mice, the strongest effect was described for the most sensitive tonotopic frequency region (16 kHz) with approx. 16-dB baseline hearing threshold shift between measurements performed under ketamine/xylazine vs. isoflurane anesthesia (Cederholm et al., 2012). A further (10-dB) increase in the hearing thresholds was observed during a 1-h long exposure to low levels of isoflurane. Similarly, an approximately 27-dB hearing threshold shift was observed in rats upon prolonged isoflurane anesthesia (Ruebhausen et al., 2012). In our experimental conditions, we also observed a rise in ABR thresholds during a 2-h exposure to isoflurane, which however was less pronounced (approx. 5–8 dB SPL) and similar across all measured frequencies (Figure 1B). It has to be noted that hearing thresholds under isoflurane anesthesia may be very sensitive to small perturbations in local anesthetic concentrations determined by the isoflurane % vol. settings and the flow of anesthetics through the system (t.i. the speed of isoflurane delivery and removal from the mask), which, in addition to animal age or deviations in other parameters can account for some of the differences observed across studies. Compared to data acquired in mice of matching ages and background, the ABR thresholds under ketamine/xylazine anesthesia are typically approx. 10 dB lower in the 8–16 kHz range as compared to pre-exposure controls in the present study, measured right after isoflurane induction (see, e.g., WT controls in Oestreicher et al., 2024 vs. controls in the current study). As demonstrated, noise exposure under isoflurane anesthesia evokes smaller hearing threshold shifts with more recovery and lower hair cell loss (Kim et al., 2005) as compared to exposure in awake animals. While we do not have data from awake exposures under otherwise identical experimental conditions, based on our data of *in vitro* isoflurane experiments and afore mentioned *in vivo* data, we believe that isoflurane anesthesia resulted in partial protection against hearing loss.

### Delayed and moderate auditory synaptopathy not accompanied by whole-cell membrane capacitance changes

Previous reports in awake exposed CBA mice demonstrated that noise-induced loss of ribbons and synapses occurs very quickly and is detected to almost full extent immediately after exposure (e.g., Liberman et al., 2015). Similarly, some immediate loss of ribbons or ribbon synapses was also detected immediately after noise-exposure in C57Bl/6J mice (Kim et al., 2019). In contrast, our data showed no loss of ribbons or ribbon synapses immediately after noise exposure apart from a slight tendency toward reduced synapses numbers for 96-dB group. The differences in the postsynaptic immunosignal could potentially be explained by the use of different immunohistochemical markers: while postsynaptic glutamate receptors may undergo internalization, an antibody against a scaffold protein Homer1, as used in the present study, may more faithfully report the presence and location of the postsynaptic boutons. However, this cannot account for the discrepancy observed presynaptically. In this aspect, our data are consistent with the observations in 6-week-old C57Bl/6J mice (of both sexes) exposed un-anesthetized to 90-dB 8-16 kHz noise band for 2-h, also detecting no ribbon loss immediately after exposure (Kaur et al., 2019). Several factors may affect the outcome of noise trauma, including noise paradigm, species, strain, age, sex, anesthesia (reviewed in Hickox et al., 2017), likely also dictating the speed of initial and likely very dynamic changes in the first hours following noise exposure.

In our experimental conditions, increasing noise levels from 92 to 96 dB did not further increase synaptopathic loss. This is consistent with recent data obtained in C57Bl/N mice exposed under reversible anesthetic (Blum et al., 2024). It is possible that larger (and possibly faster) acute damage (e.g., to stereocilia) upon higher noise levels effectively reduces the amount of hair cell activation during noise exposure and thus prevents further, more extensive excitotoxicity and there may be conditions, where this effect prevails. The idea that OHC damage may protect the IHC synapses has also been introduced in earlier studies (e.g., Fernandez et al., 2015; Liberman et al., 2015). Interestingly, while in the study by Blum et al. (2024) a larger loss of ribbons as compared to post-synapses was observed, in our experimental conditions the loss of ribbons scaled with the loss of ribbon synapses.

While IHC active zones are reportedly heterogeneous (Frank et al., 2009; Moser et al., 2023; Neef et al., 2018; Ohn et al., 2016; Özçete and Moser, 2021) and possibly a mixed (Jeffers et al., 2021; Suthakar and Liberman, 2021) rather than homogeneous subpopulation of active zones (with primarily large ribbons and large exocytic responses; Kujawa and Liberman, 2009; Özçete and Moser, 2021) may be initially lost upon noise trauma, a loss of approx. 20–23% of ribbons would still be expected to manifest as significant exocytic loss. However, this was not observed in our data. In fact, we detected a slight tendency toward increased exocytic efficiency in the basal IHCs of noise-traumatized mice. Our finding in the basal IHCs 2 weeks after noise exposure is consistent with the results obtained in young apical IHCs a day after exposure to noise evoking a transient ribbon loss (Boero et al., 2021), thus suggesting long-term effects of noise trauma on presynaptic IHC function. While we can at present not exclude further (partial) recovery of ribbons at later time points in our experimental conditions, ribbons and ribbon synapses typically do not recover considerably beyond 2 weeks post-exposure (e.g., Jeffers et al., 2021; Kujawa and Liberman, 2009; Wu et al., 2019). The observed adaptive presynaptic IHC changes might very well fit into the observation of an overall increase in ribbon size after noise trauma (Blum et al., 2024; Boero et al., 2021; Kim et al., 2019; Liberman et al., 2015; Paquette et al., 2016; Wu et al., 2019) or multiplication of ribbons in a subpopulation of active zones not distinguishable under confocal microscopy (Lu et al., 2024; Moverman et al., 2023) as well as the observation of “hyperactive” auditory nerve fibers with higher peak- and also steady-state spiking rates in the synaptopathic regions of noise-traumatized CBA-mice (Suthakar and Liberman, 2021). Ribbon multiplication and hypertrophy is expected to result in an increase in the available synaptic vesicles per active zone, which would increase IHC membrane capacitance responses, but possibly also underlie noise-induced hyperactivity of a subpopulation of auditory nerve fibers (Moverman et al., 2023; Suthakar and Liberman, 2021). Ribbon reorganization upon noise trauma was shown to be very dynamic and complex (Moverman et al., 2023; Paquette et al., 2016); in addition to ribbon size, noise trauma further affects the exact positioning of synapses within the IHCs (Liberman et al., 2015; Paquette et al., 2016). Whereas the loss of ribbons was shown to require glutamatergic neurotransmission (Hu et al., 2020; e.g., Kim et al., 2019), noise-induced remodeling of ribbons seems to be glutamate-independent (Boero et al., 2021; Kim et al., 2019). Ribbon hypertrophy may represent a form of presynaptic homeostatic plasticity and was also observed in synaptic mutants with reduced synaptic release or (local) presynaptic calcium signal (Eckrich et al., 2019; Oestreicher et al., 2024). Further work is required to understand the mechanisms that drive ribbon dynamics upon noise exposure and beyond, possibly utilizing live imaging of ribbons in various conditions (Ismail Mohamad et al., 2024; Voorn et al., 2024).

The observations of this and prior studies suggest that the hyperactivity observed in higher centers of the auditory pathways may at least partially be related to changes in the auditory periphery, particularly at the first auditory synapse. These peripheral changes could contribute to the development of hyperacusis and tinnitus in noise-exposed ears by increasing central gain in the auditory pathway (Chambers et al., 2016; Mulders and Robertson, 2013; Salvi et al., 1990). However, the adaption processes may be insufficient to fully compensate for deficits in precise sound encoding in noisy environments (Bakay et al., 2018; Monaghan et al., 2020).

In the future, noise-induced alterations in synaptic function at the level of individual IHC synapses need to be investigated to understand how heterogeneous synapses (mal-)adapt to noise-induced damage of the IHC release machinery and which of the changes observed in SGN firing may originate presynaptically.

### Acute effects of isoflurane on HC physiology and beyond

Isoflurane as a volatile gas anesthetic has many advantages compared to other commonly used anesthetics that need to be administered peritoneally, e.g., the ease of administration, consistency in the level of anesthesia and less animal handling during experiments. Isoflurane affects hearing function by increasing ABR thresholds and latencies and decreasing compound action potentials (Bielefeld, 2014; Stronks et al., 2010). In line with data from Cederholm et al. (2012), we suggest that this is not due to isoflurane effects on cochlear amplification but due to its action on synaptic calcium channels.

Traumatic noise exposure under isoflurane anesthesia leads firstly to less hearing loss and also to less damage to hair cells compared to the same noise exposure in awake animals (Chung et al., 2007; Kim et al., 2005). One proposed mechanism for this protective effect is the antagonistic effect of isoflurane on *N*-methyl-d-aspartate (NMDA) receptors, which may reduce the production of reactive oxygen species (ROS) associated with hearing loss (Chung et al., 2007; Kim et al., 2005). This hypothesis is supported by evidence of similar protective effects with other NMDA receptor antagonists (Chen et al., 2001; Ohinata et al., 2003). On the other hand, there is no evidence that ketamine can elicit such a protective effect although the drug mainly acts as an NMDA antagonist (Harada et al., 1999; Zorumski et al., 2016).

While different mechanisms of isoflurane action on various receptor types, e.g., Glycine-, GABA-, Kainate-, and NMDA-receptors are known (Dildy-Mayfield et al., 1996; Downie et al., 1996; Franks, 2008; Harrison et al., 1993), *in vitro* studies demonstrated an inhibition of the release machinery in hippocampal neurons with 1 mM isoflurane (Herring et al., 2009), and attenuation of voltage-gated sodium and calcium channels (Hemmings, 2009; Westphalen et al., 2011). Additionally, isoflurane inhibited glutamate release by reducing calcium influx and not by interfering with stimulus-vesicle-secretion coupling in hippocampal neurons (Baumgart et al., 2015; Herold et al., 2017). To investigate the effect on the calcium channels of inner hair cells, we performed patch clamp experiments on isolated hair cells treated with a clinically relevant concentration of 0.6 mM isoflurane. In the experiments we measured a reduction in calcium current amplitude and a trend toward reduced calcium-dependent exocytosis, analogous to the experiments with hippocampal neurons (Baumgart et al., 2015). Although we lack a direct comparison, reduced calcium currents and reduced glutamate transmission could be another factor how isoflurane could protect against excessive noise damage, a notion that should be tested in the future.

## Conclusion

In this study we report for the first time how a noise-induced synaptopathy affects hair cell synaptic function in the most vulnerable part of the cochlea, typically affected by noise trauma. While we did not observe an immediate loss of synapses, this was significant and amounted to approximately 25–36% 2 weeks after exposure in the high-frequency regions of the cochlea (and 11–20% in the mid-cochlea). Given the expectation that the amount of synaptic exocytosis should scale with the number of presynaptic ribbons but in line with the results recently observed in a different study that probed the presynaptic function early after noise exposure, calcium current amplitudes and calcium-dependent exocytosis remained unaffected up to 200-ms depolarizations. These results suggest compensatory presynaptic mechanisms that enhance IHC exocytosis at the remaining ribbon synapses.

While our noise exposure recordings cannot be directly compared to experiments performed in awake animals, there might be a protective effect of isoflurane on the amount of damage inflicted by noise as shown in earlier studies. The finding of an 18% decrease in IHC calcium currents due to clinically-relevant isoflurane concentration may suggest an advantage for isoflurane and similar inhalation anesthetics during otological and neurosurgical procedures involving drilling of the skull and in particular the temporal bone, as they may provide some, but not complete, protection from acoustic trauma. While drilling of the skull is typically significantly shorter (20–45 min, longer in case of more complicated cases), it may be louder than noise levels used in this study and can easily exceed 100 dB SPL (Kylén et al., 1977; Yin et al., 2011), in particular when drilling on the ossicular chain or during cochleostomy in temporal bone studies (Jiang et al., 2007; Pau et al., 2007; Yu et al., 2014), and even when drilling cortical bone (Yin et al., 2011). The amount of noise exposure may vary between ear surgeries, depending on the location of drilling, the type of and the pressure applied to the drill, nevertheless, possible noise damage to the ear should always be taken into consideration (Banakis Hartl et al., 2017; Kylén et al., 1977; Parkin et al., 1980). The choice of anesthesia may influence the risk of noise-induced damage, with inhalation anesthesia possibly offering some protection.

## Data Availability

The raw data supporting the conclusions of this article will be made available by the authors, without undue reservation.
