# Supplementary material for: Noise-induced ribbon synapse loss in the mouse basal cochlear region does not reduce inner hair cell exocytosis
Source: Front Cell Neurosci. 2025 Jan 7;18:1523978. doi: 10.3389/fncel.2024.1523978 (PMC11747652; doi:10.3389/fncel.2024.1523978)
Supplement: Supplementary file 1 [file Image_1.PDF]

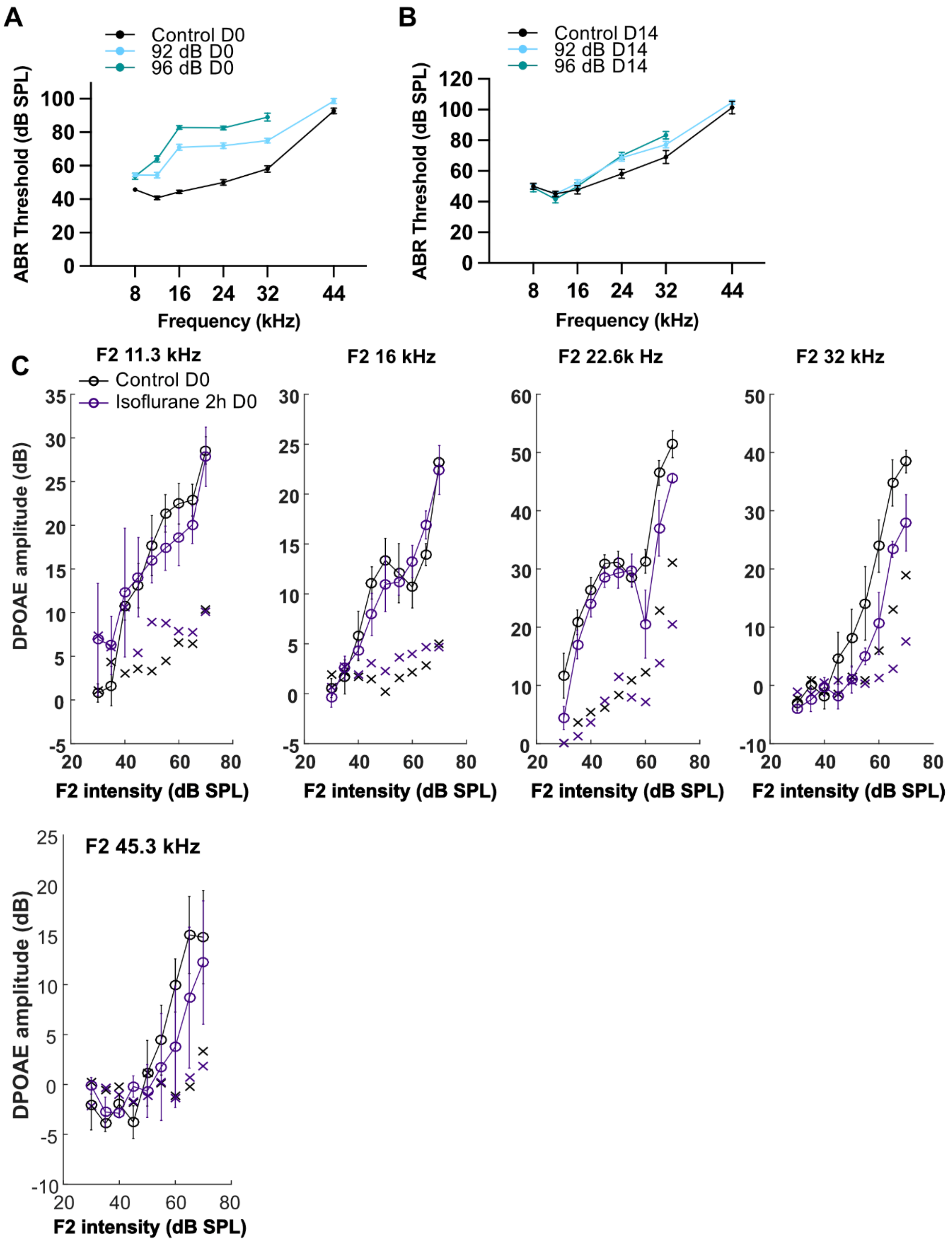

Figure S1. Systems physiology upon exposure to noise or prolonged isoflurane anesthesia

(A-B) ABR Thresholds upon noise trauma with 92 and 96 dB noise bands at D0 (A) and D14 (B). **(C)** Amplitudes of DPOAE-recordings are unaffected upon prolonged isoflurane exposure (N=4 for Control D0 and Isoflurane 2h D0).

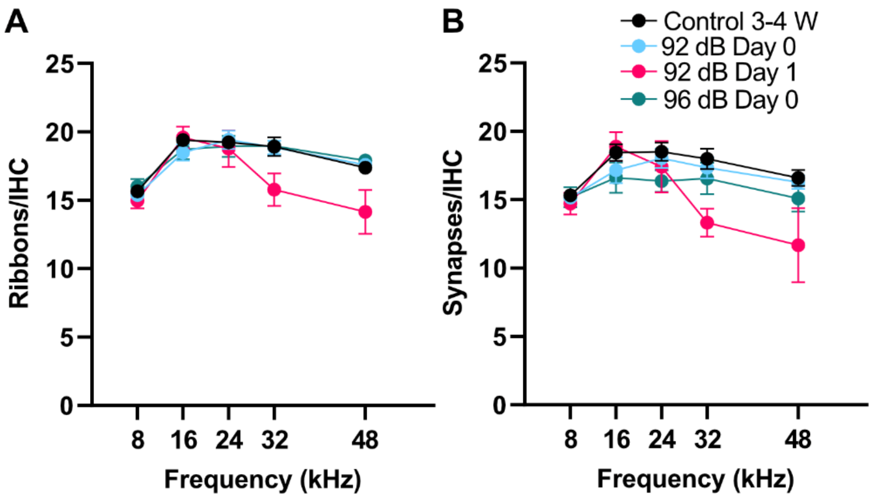

Figure S2. IHC ribbon and ribbon synapse counts immediately after and one day after noise exposure

The number of IHC ribbons **(A)** and ribbon synapses **(B)** is unchanged immediately after, but declines one day after noise trauma. On D1, a significant loss of 17-19% of ribbons and 25-30% of ribbon synapses (t.i. juxtaposed CtBP2/Homer1 immunospots) in the high-frequency regions (32-48kHz) was observed (Dunnett's test; Control D0 vs. 92 dB D1: $p < 0.01$ for 32 and 44 kHz; 92 dB D1 N = 4, 5, 5, 5, 5).
