# Supplementary material for: Noise-induced ribbon synapse loss in the mouse basal cochlear region does not reduce inner hair cell exocytosis
Source: Front Cell Neurosci. 2025 Jan 7;18:1523978. doi: 10.3389/fncel.2024.1523978 (PMC11747652; doi:10.3389/fncel.2024.1523978)
Supplement: Supplementary file 2 [file Table_1.DOCX]

**Table S1.** **Statistical analysis of the abundance of ribbons and synapses in the IHCs of different cochlear tonotopic regions**. The table lists the adjusted *p*-values of the Dunnett’s multicomparisons tests upon two-way ANOVA for the synapse and ribbon counts. Statistically significant *p*-values are represented by asterisks: *p* < 0.05 (*), *p* < 0.001 (***), *p* < 0.0001 (****).

| **Ribbons D0** | | | | |
| --- | --- | --- | --- | --- |
| Adjusted *p*-values | | Control D0 vs. 92 dB D0 | Control D0 vs. 96 dB D0 |  |
| **Tonotopic region** | **8 kHz** | 0.90 | 0.84 |  |
|  | **16 kHz** | 0.33 | 0.56 |  |
|  | **24 kHz** | 0.96 | 0.90 |  |
|  | **32 kHz** | 1.00 | 1.00 |  |
|  | **48 kHz** | 0.96 | 0.73 |  |
| **Ribbons D14** | | | | |
| Adjusted *p*-values | | Control D14 vs. 92 dB D14 | Control D14 vs. 96 dB D14 | Control D14 vs. Isoflurane 2h D14 |
| **Tonotopic region** | **8 kHz** | 1.00 | 0.78 | 0.93 |
|  | **16 kHz** | 0.60 | 0.84 | 0.95 |
|  | **24 kHz** | 0.08 | 0.40 | 1.00 |
|  | **32 kHz** | **** | *** | 0.78 |
|  | **48 kHz** | **** | * | 0.30 |
| **Synapses D0** | | | | |
| Adjusted *p*-values | | Control D0 vs. 92 dB D0 | Control D0 vs. 96 dB D0 |  |
| **Tonotopic region** | **8 kHz** | 0.98 | 0.99 |  |
|  | **16 kHz** | 0.33 | 0.12 |  |
|  | **24 kHz** | 0.86 | 0.06 |  |
|  | **32 kHz** | 0.76 | 0.31 |  |
|  | **48 kHz** | 0.95 | 0.28 |  |
| **Synapses D14** | | | | |
| Adjusted *p*-values | | Control D14 vs. 92 dB D14 | Control D14 vs. 96 dB D14 | Control D14 vs. Isoflurane 2h D14 |
| **Tonotopic region** | **8 kHz** | 0.99 | 0.47 | 0.98 |
|  | **16 kHz** | 0.89 | 0.06 | 0.99 |
|  | **24 kHz** | * | * | >0.99 |
|  | **32 kHz** | **** | **** | 0.81 |
|  | **48 kHz** | **** | *** | 0.69 |
